# A potent inflammatory response is triggered in asymptomatic blood donors with recent SARS-CoV-2 infection

**DOI:** 10.1590/0037-8682-0239-2022

**Published:** 2022-10-21

**Authors:** Marina Lobato Martins, Maria Clara Fernandes da Silva-Malta, Argus Leão Araújo, Fabíola Araújo Gonçalves, Maiara de Lourdes Botelho, Isabelle Rocha de Oliveira, Luciana de Souza Madeira Ferreira Boy, Hélinse Medeiros Moreira, Edel Figueiredo Barbosa-Stancioli, Maísa Aparecida Ribeiro, Daniel Gonçalves Chaves

**Affiliations:** 1 Fundação Centro de Hematologia e Hemoterapia de Minas Gerais/Hemominas, Belo Horizonte, MG, Brasil.; 2 Fundação Hospitalar do Estado de Minas Gerais, Hospital Eduardo de Menezes, Belo Horizonte, MG, Brasil.; 3 Universidade Federal de Minas Gerais, Instituto de Ciências Biológicas, Departamento de Microbiologia, Laboratório de Virologia Básica e Aplicada, Belo Horizonte, MG, Brasil.

**Keywords:** SARS-CoV-2, COVID-19, Cytokines, Chemokines, Asymptomatic

## Abstract

**Background:**

The inflammatory response plays a significant role in the outcome of coronavirus disease (COVID-19).

**Methods:**

We investigated plasma cytokine and chemokine concentrations in non-infected (NI), asymptomatic severe acute respiratory syndrome-coronavirus-2 (SARS-CoV-2)-infected blood donors (AS), and patients with severe COVID-19 (SC).

**Results:**

The SC group showed significantly higher levels of interleukin 6 (IL-6), IL-10, and CCL5 than the AS and NI groups. The SC and AS groups had considerably greater CXCL9 and CXCL10 concentrations than the NI group. Only NI and infected people showed separate clusters in the principal component analysis.

**Conclusions:**

SC, as well as AS was characterized by an inflammatory profile.

Patients with severe acute respiratory syndrome coronavirus 2 (SARS-CoV-2) infection present diverse initial symptoms, with the most common symptoms being influenza-like. Disease progression can lead to worsening respiratory symptoms, such as dyspnea and hypoxemia, and in severe cases, acute respiratory distress syndrome and septic shock can be fatal^1^. SARS-CoV-2 infection triggers an immune reaction that can lead to a significant change in the leukocyte count as well as an increase in the production of cytokines^2^. High production and systematic circulation of cytokines, known as cytokine storm, has been identified as one of the most important mechanisms underlying disease progression and coronavirus disease (COVID-19) severity^2^. However, the mechanisms by which cytokine storm and leukocyte changes affect the outcome of COVID-19 remains unknown, although they may be protective or lethal in high- and low-risk individuals. Therefore, the search for risk biomarkers has been of great interest. 

We investigated 31 patients with severe COVID-19 (SC) who visited the Hospital Eduardo de Menezes, FHEMIG (Fundação Hospitalar do Estado de Minas Gerais) between July and November 2020, when their samples were collected. SARS-CoV-2 infection was confirmed in swab samples via reverse transcription-polymerase chain reaction (RT-PCR) assays performed at the State Central Laboratory (Fundação Ezequiel Dias). The median age of the patients was 60 years [interquartile range (IQR), 55-65] and a majority (18/31, 58.1%) were female. We also analyzed the data of 24 asymptomatic blood donors (AS) with active SARS-CoV-2 infection, which was identified in saliva samples by RT-PCR as previously described^3^. Testing for anti-SARS-CoV-2 IgG (chemiluminescence microparticle immunoassay; Abbott Ireland Diagnostics, Sligo, Ireland) yielded negative results for 87.5% of these subjects, indicating recent infection. These participants were enrolled during blood donation at the Hemocentro de Belo Horizonte, Fundação Hemominas, between June and September 2020. They were all eligible to donate blood and remained asymptomatic or developed a mild disease after blood donation. In addition, 36 non-infected (NI) blood donors whose samples were collected between March and December 2020 and tested negative for anti-SARS-CoV-2 IgG were included for comparison. The AS group consisted of 11 (46%) males and 13 (54%) females, with a median age of 28 years (IQR 27.1-33.6), whereas the NI group comprised 25 (69%) males and 11 (31%) females, with a median age of 36 years (IQR 31.8­-38.6). The study protocol was approved by the ethics committees of the participating institutions (approval nº CAAE 31087720.2.0000.5118 and 31087720.2.3002.5124).

Plasma cytokine and chemokine concentrations were measured using blood samples collected from participants in EDTA tubes. The Human Th1/Th2/Th17 Cytokine CBA kit (Becton Dickinson, San Jose, CA, USA) was used to quantify interleukin 2 (IL-2), IL-4, IL-6, IL-10, tumor necrosis factor (TNF), interferon γ (IFN-γ), and IL-17A. The Chemokine CBA kit (Becton Dickinson) was used to quantify CCL2 (MCP-1, monocyte chemoattractant protein-1), CCL5 (RANTES, regulated on activation, normal T cell expressed and secreted), IL-8 (CXCL8), CXCL9 (MIG, monokine induced by gamma interferon), and CXCL10 (IP10, interferon gamma-induced protein 10). The assay was performed according to the manufacturer’s instructions. The fluorescence from the labeled beads was captured by Accuri C6 flow cytometer (Becton Dickinson, Franklin Lakes, NJ, USA), using 1,800 events/sample and 300 beads/biomarker. The data were analyzed using FCAP Array software version 3.0 (Becton Dickinson). Proportion analyses of categorical variables were performed using Fisher’s exact test. For continuous variables, the Kruskal-Wallis test was used to compare all groups, and when the null hypothesis was rejected, we applied a post hoc Dunn’s multiple comparison test. The Mann-Whitney *U* test was used for pairwise comparisons. The significance level was set at 5%. Multivariate analysis of the concentrations of cytokines and chemokines for cluster evaluation (principal component analysis and heatmap) was performed using the ClustVis virtual tool (http://biit.cs.ut.ee/clustvis). 

A majority (90.3%) of COVID-19 patients had at least one comorbidity, with hypertension (71.0%) being the most frequent, followed by diabetes (51.6%) and obesity (25.8%). When patients were hospitalized, cough, dyspnea, fever, and myalgia were the most common COVID-19 symptoms, followed by fatigue, sputum production, headache, and anosmia. The median time from symptom onset to hospital admission was 7 days (range 2-31). Fourteen (45.2%) patients required intensive care unit (ICU) care, lasting for an average of seven days. There were no statistically significant differences between the groups in terms of the frequency of comorbidities and the frequency of COVID-19 signs and symptoms on hospital admission when comparing patients who required (ICU) or did not require (non-ICU) intensive care. Supplemental oxygen was used by most non-ICU patients (88.2%) and all ICU patients, with no statistical difference between the two groups. However, the ICU group required almost twice the average hospitalization time compared to the non-ICU group (19 vs. 10 days, p<0.016). COVID-19 was not fatal in any patient. There was a significant difference in sex proportions between the SC and NI groups (p=0.029). Patients in the SC group were significantly older than those in the NI and AS groups (p<0.001). 

Univariate analysis (Table 1) showed significant differences in the plasma concentrations of IL-2, IL-4, IL-6, IL-10, IFN-γ, CCL5, CXCL8, CXCL9, and CXCL10 between specific groups. The concentrations of IL-4 and CXCL8 were significantly higher in NI than in AS subjects, whereas AS subjects had significantly higher levels of IFN-γ, CCL5, CXCL9, and CXCL10. When the NI and SC groups were compared, even more differences were observed. Compared to NI subjects, COVID-19 patients had significantly elevated levels of IL-6, IL-10, IFN-γ, CCL5, CXCL9, and CXCL10 and decreased levels of CXCL8. Although elevated CXCL8 production has been reported in patients with COVID-19^4^, steroid treatment can rapidly reduce the concentration of CXCL8, CCL2, and CXCL10^5^, which could contribute to the lower concentration of these chemokines recorded in these patients compared to AS and/or NI subjects, since most of them (29/31, 93.5%) received corticosteroids as part of their treatment.

**TABLE 1:** Concentrations of plasma cytokines and chemokines in samples from noninfected individuals, asymptomatic infected individuals, and patients with severe COVID-19.

Biomarker level, pg/mL	NI	AS	SC patients						
	(n=36)	(n=24)	All	ICU	Non-ICU	p-value			
			(n=31)	(n=14)	(n=17)	NI × AS	NI × All	AS × All	ICU × non-ICU
**IL-2**	0.11	0.14	0.00	0.27	0.00	ns			0.061
**IL-4**	0.41	0.10	0.20	0.22	0.20	**s**	ns	ns	0.776
**IL-6**	0.67	1.02	2.24	2.87	1.60	ns	**s**	**s**	0.364
**IL-10**	0.00	0.15	0.68	0.88	0.44	ns	**s**	**s**	0.536
**TNF**	0.00	0.00	0.00	0.00	0.00	ns			0.778
**IFN-γ**	0.00	0.37	0.00	0.25	0.00	**s**	**s**	ns	**0.027**
**IL-17A**	0.68	0.50	0.42	0.81	0.31	ns			0.421
**CXCL8**	8.29	0.00	0.00	0.00	0.00	**s**	**s**	ns	0.857
**CCL2**	26.52	18.8	13.73	12.64	16.73	ns	**s**	ns	0.764
**CCL5**	2523	3404	6273	6014	6374	**s**	**s**	**s**	0.766
**CXCL9**	10.83	98.83	115.5	176.1	94.73	**s**	**s**	ns	0.137
**CXCL10**	27.69	788.7	566.6	433.2	613.0	**s**	**s**	ns	0.620

**NI:** Noninfected blood donors, negative for anti-SARS-CoV-2 IgG; **AS:** Asymptomatic blood donors during sample collection who tested RT-PCR positive for SARS-CoV-2; **SC:** Patients with severe COVID-19 at the time of blood collection (SARS-CoV-2 infection confirmed via RT-PCR); **ICU or non-ICU:** Patients with severe COVID-19 who required or did not require intensive care during hospitalization, respectively. The values are presented as median concentrations. Statistical analysis was performed using the Kruskal-Wallis test for the combined comparison of NI, AS, and All patients, followed by Dunn’s multiple comparison test. Significant differences are indicated as “s” and non-significant differences as “ns”. ICU and non-ICU patients were compared using the Mann-Whitney *U* test.

In turn, in comparing the SC and AS groups, only IL-6, IL-10, and CCL5 persisted at significantly higher levels in patients. In comparing the SC subgroups, only IFN-γ levels showed a significant difference and were higher in ICU patients than in non-ICU patients. Apart from the small sample size, the absence of other differences may be due to the clinical similarity between non-ICU and ICU patients. 

Given that the ratio of pro- to anti-inflammatory cytokine levels may be more important in determining the type of immune response, we analyzed the ratios of IL-6/IL-10 and IFN-γ/IL-10 in the SC and AS groups. IL-6 was detected in all individuals in both groups. IFN-γ and IL-10 were detected in 58.3% (14/24) of AS subjects, and 41.9% (13/31) and 77.4% (24/31) of SC patients, respectively. Comparing individuals with detectable concentrations, there was no significant difference in the IL-6/IL-10 ratio between the AS and SC groups (p=0.964); however, the AS group showed significantly higher IFN-γ/IL-10 ratios than the SC group (p=0.031). This result suggests that IFN-γ may play an important role in the onset response against the virus to control its spread throughout the body. In line with this finding, Chen et al. discovered that IFN-γ expression by CD4+ T cells was lower in severe cases than in moderate cases^6^.

To confirm the clustering of subjects based on cytokine and chemokine concentrations, multivariate analysis using principal component analysis (PCA) and a heatmap was performed. PCA showed that the levels of these biomarkers were able to distribute the NI group and SARS-CoV-2 infected individuals into clearly distinct clusters, whereas the AS group was homogenously distributed in a cluster overlapping that formed by SC, whose ICU and non-ICU patients were not grouped differently (Figure 1). Heatmap analysis showed the profile of these groups, highlighting the high levels of CCL5, CXCL9, and CXCL10 in individuals infected with SARS-CoV-2 (Figure 2). CCL5 levels were elevated in infected individuals, reaching their highest levels in patients who developed severe disease. CCL5 is expressed early and can activate cells directly involved in antiviral responses, such as NK cells, T CD4+ lymphocytes, monocytes, mast cells, and dendritic cells^7^. Elevated levels of CCL5 have been observed in COVID-19 patients compared to noninfected individuals; however, published data have not been concordant between infected individuals with different clinical outcomes^8^ ^,^ ^9^. 

**FIGURE 1:**
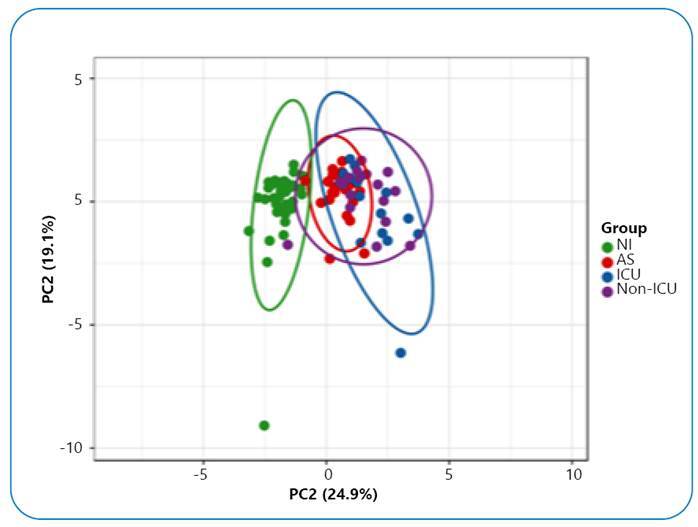
Principal component analysis of cytokines and chemokines in noninfected (NI, negative for anti-SARS-CoV-2 IgG), asymptomatic (AS, positive on RT-PCR) blood donors, and severe COVID-19 patients who required (ICU) or did not require (non-ICU) intensive care. The X and Y axes show principal component 1 (PC1) and principal component 2 (PC2), respectively, explaining the proportion of the total variance. Unit variance scaling was applied and singular value decomposition with imputation was used to calculate principal components. Ellipses represent the 95% probability that a fresh observation from the same group will fall inside the ellipse.

**FIGURE 2:**
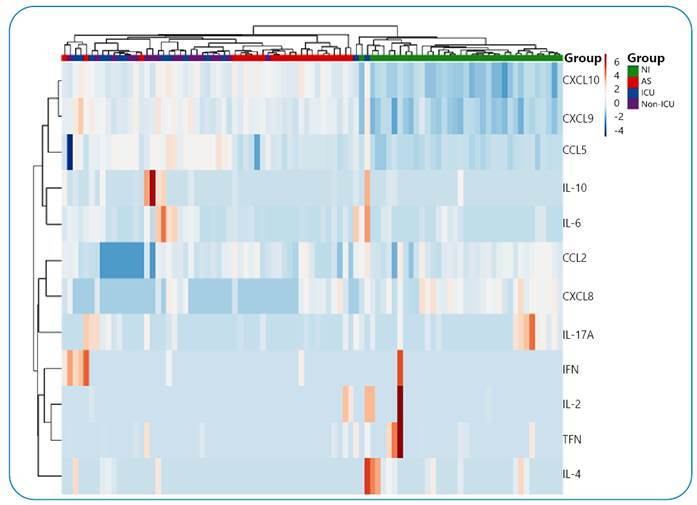
Heatmap of cytokine and chemokine profiles in noninfected (NI, negative for anti-SARS-CoV-2 IgG), asymptomatic (AS, positive on RT-PCR) blood donors, and severe COVID-19 patients who required (ICU) or did not require (non-ICU) intensive care. Original values are ln(*x*)-transformed. Rows are centered; unit variance scaling was applied to rows. Correlation distance and average linkage are used to cluster both rows and columns. Tree ordering had a higher median value first.

CXCL9 and CXCL10 are chemokines with high concentrations in infected individuals. CXCL9 and CXCL10 are known to play a crucial role in the human antiviral response, and their concentrations have been previously related to COVID-19 infection and its severity^10^. We previously found a significant strong or moderate inverse correlation between the neutralizing capacity of anti-SARS-CoV-2 IgG antibodies and the levels of CCL5, CXCL9, and CXCL10^11^, suggesting that humoral responsiveness influences the chemokine production network.

Age can also influence the production of inflammatory markers. Weak to moderate positive correlations between age and some biomarkers were observed in the SC (IL-6: *r*=0.508, p=0.004; IFN: *r*=0.356, p=0.049; CXCL8: *r*=0.504, p=0.004; CXCL9: *r*=0.403, p=0.025) and NI (CXCL9: *r*=0.537, p<0.001; CXCL10: *r*=0.416, p=0.012) groups. These correlations seem to be related to an age threshold, since most of them were observed only in the SC group, which comprised older people.

Several studies have analyzed different groups of individuals infected with SARS-CoV-2 to identify biomarkers associated with infectious outcomes. Liu et al. reviewed 55 studies that compared patients with severe and non-severe COVID-19 and found that the serum levels of IL-2, IL-2R, IL-4, IL-6, IL-8, IL-10, and TNF-α were significantly upregulated in patients with severe disease, with a sharp difference for IL-6 and IL-10^12^. Another systematic review presented data from studies that showed changes in circulating leukocyte subsets and cytokine secretion in patients with mild and severe forms of the disease, particularly IL-6, IL-1β, IL-10, TNF, GM-CSF, CXCL10, IL-17, MCP-3, and IL-1ra^2^. The authors posited that there is a lack of consensus regarding cut-off values of cytokine-based biomarkers that could indicate disease progression. Non-concordant data on increased or decreased levels of immune response markers in COVID-19 patients with varying clinical states have been described. Levels of immune response biomarkers in SARS-CoV-2 infection may vary depending on different factors, such as age, type and frequency of comorbidities, disease severity, treatment, and duration of infection on sample collection. Because of this, it is necessary to share findings from a broad range of investigative approaches.

In our study, elevated levels of IL-6, IL-10, and CCL5 seemed to be remarkable biomarkers for differentiating patients with severe COVID-19 from AS and NI patients, suggesting an association with progression to severe COVID-19. Several studies have consistently found an association between high plasma concentrations of IL-6 and IL-10 and increased COVID-19 severity^10^. IL-6 is very important in the early immune response to infections and mediates the uncontrolled production of chemokines and cytokines. In addition, highly increased IL-6 concentrations can be decisive in severe inflammatory conditions in COVID-19^13^. IL-10 and IL-4 are known to play important roles in the regulatory and anti-inflammatory responses. The increased production of IL-10, but not of IL-4, in severe COVID-19 suggests that IL-10 is the main cytokine that counteracts the inflammatory response cascade triggered by the Th1 type response. 

It was very interesting to observe in the PCA that despite having very recent infection, AS individuals were clustered and nested in the set of patients with severe COVID-19, with no overlap with the NI group, which provides evidence of the occurrence of a potent inflammatory response against the virus, even in the absence of symptoms. This raises the issue that, under pandemic conditions, the risk of producing blood components from eligible asymptomatic infected donors with an inflammatory profile is increased. Although there is no evidence that SARS-CoV-2 can be transmitted by blood transfusion^3^, it is recognized that pro-inflammatory cytokines accumulated in blood products may cause transfusion-associated adverse reactions and transfusion-induced systemic inflammation^14^ ^,^ ^15^. In the same way, blood components from unrecognized infected blood donors may pose an additional risk for adverse transfusion events, particularly among critically-ill patients in whom elevated levels of inflammatory molecules are associated with poor outcomes. Therefore, it is important to verify whether high levels of inflammatory modulators accumulated in blood components from SARS-CoV-2 infected blood donors can increase the risk of adverse transfusion events. 

This study has some limitations. First, the number of patients enrolled was small. The time of sample collection after symptom onset or hospital admission differed among patients, and longitudinal samples were not analyzed. Age differences, in addition to other factors not investigated, such as virus variants and the genetic background of the individuals, can influence the immune response and cytokine production, limiting the findings of this study.

In conclusion, both asymptomatic SARS-CoV-2 infected blood donors and patients with severe COVID-19 share an inflammatory profile; however, high levels of IL-6, IL-10, and CCL5 were significantly associated with severe infection outcomes. Hemovigilance measures for adverse transfusion events should be reinforced in situations such as the COVID-19 pandemic, in which a high percentage of unrecognized asymptomatic infected individuals could be eligible to donate blood.
